# What I missed from my online therapist: A survey-based qualitative investigation of patient experiences of therapist contact in guided internet interventions

**DOI:** 10.3389/fpsyg.2023.990833

**Published:** 2023-02-02

**Authors:** Hanna Sayar, Jon Vøllestad, Tine Nordgreen

**Affiliations:** ^1^Department of Mental Health and Addiction, Oslo University Hospital, Oslo, Norway; ^2^Department of Clinical Psychology, Faculty of Psychology, University of Bergen, Bergen, Norway; ^3^Division of Psychiatry, Haukeland University Hospital, Bergen, Norway; ^4^Department of Global Public Health and Primary Care, Faculty of Medicine, University of Bergen, Bergen, Norway

**Keywords:** guided ICBT, internet, depression, anxiety, therapist-assisted, qualitative content analysis

## Abstract

**Background:**

The effectiveness of internet-delivered cognitive behavioral therapy (ICBT) in alleviating symptoms of psychological disorders has been demonstrated across qualitative and quantitative studies. Generally, guided ICBT is considered more effective than unguided ICBT. Yet, what therapist contact and guidance specifically add to the treatment is less clear. There is a need for more knowledge about how patients experience the relationship with their therapist in guided ICBT. The aim of the study was to explore what patients missed in the contact with their therapist in guided ICBT in routine care.

**Methods:**

The study used a qualitative design to explore patients´ experiences of the therapist contact in guided ICBT for social anxiety disorder, panic disorder and major depressive disorder. Following treatment, 579 patients received a survey with the open-ended question “What did you miss in the contact with your therapist?” The responses were explored thematically using qualitative content analysis.

**Results:**

A total of 608 unique responses were provided. Of these, 219 responses gave voice to some degree of perceived lack or limitation in their interaction with the therapist or the treatment in general. The analysis yielded three main categories: The first theme, Therapist-ascribed shortcomings, concerned experiences of something missing or lacking in the contact with the ICBT therapist. More specifically, the patients expressed a need for more emotionally attuned and tailored interaction. The second theme was Program obstacles, encompassing expressed wishes for increased therapist responsivity and more contact face-to-face. Self-attributed limitations, the third category, concerned patient experiences of barriers to treatment engagement as originating in themselves.

**Conclusion:**

This study sheds light on what patients receiving guided ICBT in routine care missed in the contact with their therapist. The patients who expressed that something was missing in the contact with their therapist constituted a small part of the responses in the sample, even after being directly asked. The themes that emerged point to significant experiences of being inadequately related and responded to, both with potential adverse consequences for the treatment. These findings give new insights to the role of the guidance in ICBT and have implications for the training and supervision of guided ICBT therapists.

## Introduction

Guided internet-delivered cognitive behavioral therapy (ICBT) is a type of manualized psychological treatments provided online through a computer or mobile phone with guidance from a therapist. ICBT includes psychotherapeutic strategies such as psychoeducation, cognitive restructuring, and exposure exercises to facilitate more adaptive ways of thinking and behaving for individuals with mental health disorders (Andersson et al., 2019). The treatment is characterized by predefined content organized in modules, where the patient receives asynchronous minimal therapist guidance regularly through secure emails (Smoktunowicz et al., 2020). The treatment is time-limited, skill-oriented and requires the patient to work systematically and independently. Guided ICBT is increasingly being suggested as a feasible and scalable treatment alternative that can increase access to evidence-based treatments (Titov et al., 2018). The clinical efficacy of guided ICBT in alleviating psychological symptoms and improving quality of life has been established across a number of randomized controlled trials (Andersson and Berger, 2021). The treatment also remains effective when implemented in routine care (Titov et al., 2018). Despite its proven clinical utility and implementation, a number of patients are not adequately helped by ICBT, and attrition rates can be high (Moshe et al., 2021). In guided ICBT, the therapist role aims to improve outcomes by facilitating the patient’s experienced relevance of and adherence to treatment. However, there is a need for more knowledge about how patients experience the interaction with their therapist during guided ICBT in routine care.

Reviews and meta-analyses indicate that therapist contact in guided ICBT matters, in that guided ICBT is generally considered to be more effective compared with unguided ICBT (Spek et al., 2007; Karyotaki et al., 2017). These findings suggest that contact with a therapist is an active component in making ICBT effective. Studies of the therapeutic alliance in guided ICBT find that a positive alliance is formed in this modality of treatment, on par with what is found for face-to-face therapy (Pihlaja et al., 2018) and with similar correlations between alliance and outcome (Probst et al., 2019; Andersson and Berger, 2021). Yet, what therapist contact and guidance specifically add to the treatment is less clear. The interaction between therapist and patients is quite distinct from face-to-face therapy both in terms of mode of communication, response latency, and amount of contact. It is therefore of relevance to understand which features of the interaction in guided ICBT that patients emphasize as important.

One line of studies has explored distinct therapist behaviors in ICBT, and their relation to outcome. Paxling et al. (2013) analyzed emails from three therapists to 44 patients that received guided ICBT for generalized anxiety disorder. They found that the therapist behavior of task reinforcement was positively associated with treatment outcome whereas deadline flexibility was negatively associated with treatment outcome. In a similar study, Holländare et al. (2016) examined emails sent from five therapists to 42 patients that completed guided ICBT for depression. The most common therapist behaviors were encouraging, affirming, guiding, and urging, where only affirming was associated with increased adherence and symptom improvement up to 2 years after treatment. These two studies point to therapist guidance being a factor related to outcome. Another line of research has examined whether the organization of interaction with the therapist within the guided ICBT program affects outcome. Examples of this include different frequencies of contact (Klein et al., 2009; Aardoom et al., 2016), prescheduled versus on-demand guidance (Hadjistavropoulos et al., 2017; Zagorscak et al., 2018; Zetterberg et al., 2019) and variations in therapist responsivity (Alfonsson et al., 2016; Hadjistavropoulos et al., 2020). Results appear to be mixed, but it seems to be the case that guided ICBT with different amounts and frequencies of therapist guidance can be equally effective. However, less is known about patient’s perceptions of the support and interaction with their therapist. Therefore, it is important to explore how patients receiving guided ICBT in routine care experience the interaction and quality of contact with their therapist.

Qualitative investigations are particularly suited to investigate patient experiences. Some studies have used in-depth interviews to explore patient perceptions of the guided ICBT treatment process. In an early study, Bendelin et al. (2011) conducted semi-structured interviews with patients that received guided or unguided ICBT for depression. The interviews revealed that those who had a practical hands-on approach to treatment, “doers,” reported more positive views of treatment and more favorable outcome than the “readers” and “strivers” who were less engaged in treatment and struggled to benefit from the treatment format. While for some participants, the treatment was seen as supportive of autonomy and self-efficacy, others missed features associated with face-to-face treatment and this seemed to be associated with reduced engagement and less favorable outcomes (Bendelin et al., 2011). Furthermore, participants who were less engaged reported being more impaired by the perceived lack of support, contact, and flexibility of the treatment program.

Several qualitative studies yield similar dimensions of patients being enabled versus struggling with engagement with the treatment format (i.e., Johansson et al., 2015; Holst et al., 2017; Patel et al., 2020; Bragesjö et al., 2021). Johansson et al. (2015) analyzed data from in-depth interviews with patients who were non-adherent to guided ICBT for generalized anxiety disorder. These patients experienced the treatment as burdensome for a number of reasons, among them being the experience that the therapist was not caring enough, as well as the expectation of face-to-face treatment as being more conducive to support and motivation (Johansson et al., 2015). Similarly, Holst et al. (2017) found that while some patients receiving guided ICBT for depression in primary care saw the treatment as a good fit, others expressed a need for a deeper and more personal online guidance, whereas others needed more face-to-face interaction. A recent study by Bragesjö et al. (2021) interviewed patients who had received guided ICBT for symptoms of post-traumatic stress. In this study, guided ICBT was on one hand seen as facilitating autonomy, and lacking sufficient support, contact in person and tailoring to individual needs on the other. A recent meta-synthesis on patient experiences of digital health interventions by Patel et al. (2020) found three main themes seen as important aspects of effective treatment; initial motivation, hopes and expectations, the tailoring of the treatment program, and the presence and support from a therapist throughout treatment. Thus, common themes from qualitative investigations of patient experiences of ICBT concern the amount and quality of contact with the therapist, as well as the structural features of the format.

In a different qualitative approach to investigating participant experiences, some studies have explored the responses of larger numbers of patients receiving guided ICBT to open-ended survey questions using content analyses. Richards et al. (2016) collected qualitative and quantitative data on user experience from 281 participants in guided ICBT for depression. A majority experienced guided ICBT as informative, user-friendly, and accessible. However, a minority wished for more personalized guidance, and some also felt their therapist to be impersonal. Similarly, Hadjistavropoulos et al. (2018) inquired 225 patients about what was liked and disliked about guided ICBT for depression and anxiety, where the negative experiences pertained to the experienced inflexibility of the treatment program and lack of contact with the therapist. Across qualitative and quantitative investigations of how patients experience guided ICBT, a distinction emerges between those who see the format as enabling, and those who for various reasons see the program as inflexible and lacking in terms of support and personal relevance.

As guided ICBT is increasingly being implemented in routine care it is important to provide high quality therapist guidance that is based on patient’s experiences and needs during treatment. The aim of the current study was to expand on the existing literature on patient experiences of the therapist role in guided ICBT. We explored what patients missed in the contact with their therapist during guided ICBT for depression, social anxiety disorder and panic disorder in routine care through a qualitative content analysis of replies on a survey that patients routinely fill out after completing treatment.

## Materials and methods

### Participants

Since 2013, the eCoping (eMeistring.no) clinic at Haukeland University Hospital, Bergen, Norway, has provided guided ICBT as part of routine secondary mental health services. The programs for panic disorder (PD) and social anxiety disorder (SAD) were implemented in 2013, whereas the program for major depressive disorder (MDD) was implemented in 2015. The clinic has a catchment area of 300,000 persons. Patients were recruited from three clinical trials investigating the effectiveness of guided ICBT in a clinical routine-care setting. All patients recruited in the current study received guided ICBT for MDD, SAD, or PD at a public secondary mental health care outpatient clinic in Bergen, Norway (Nordgreen et al., 2018a,b; Nordgreen et al., 2019). All patients who were offered guided ICBT were invited to take part in the current study. The Western Regional Committee for Medical and Health Research Ethics in Norway approved the present study (2012/2211/REK; 2015/878/REK). All included participants signed a written informed consent form.

Patients were either referred to the clinic from their general practitioner, from another secondary health care clinic or contacted the clinic directly. All patients started the treatment with a face-to-face assessment interview at the clinic, including a structured diagnostic interview with the Mini International Neuropsychiatric Interview (MINI; Sheehan et al., 1998) and an anamnestic interview. Inclusion criteria were: (1) major depression, SAD, or PD as the main problem according to the MINI, (2) 18 years of age or older, (3) not using benzodiazepines on a daily basis, (4) if using antidepressants, a stable dosage over the previous 4 weeks, and (5) able to read and write in Norwegian. The exclusion criteria were: (1) current suicidal ideation, (2) current psychosis, (3) current substance abuse, (4) in immediate need of other treatment, and (5) no access to the internet.

In total, 579 patients were included in the study where 122 patients received ICBT for major depression, 223 received ICBT for SAD, and 217 received ICBT for PD, and for 17 patients the treatment category was not specified. This constitutes the full sample of participants providing post-assessments between November 2014 and March 2019. Although the treatments differ in content, they all require similar levels of therapist engagement and contact. On average, patients were in their early 30s (*M*age = 33.58; *SD* = 11.3), and the majority of participants were female (*n* = 379; 65%).

### Intervention and procedure

The guided internet-delivered treatments were built on treatments developed and evaluated in Sweden (i.e., Andersson et al., 2005; Carlbring et al., 2006; Furmark et al., 2009; Hedman et al., 2014) and then translated and evaluated in Norway (Nordgreen et al., 2010; Nordmo et al., 2015; Nordgreen et al., 2016; Jakobsen et al., 2017). All the studies showed large effect sizes for the ICBT treatments (Nordgreen et al., 2018a,b; Nordgreen et al., 2019).

The guided ICBT program for PD, SAD and MDD consists of eight (depression) or nine (anxiety disorders) text-based modules provided online. The programs include state-of the art components of CBT tailored to the respective disorders. Treatment time is set to 14 weeks and the patients are expected to spend 7–10 days per module. After each completed module, a therapist gives feedback and guidance on the work done by the patient and then introduces the next module, all asynchronous in the secure digital platform. The therapist aims to follow the same patient throughout the treatment; however, changes may occur during holidays, sick leave etc. If the patient is not heard from for 1 week, the therapist makes contact *via* a secure message and mobile phone text-message to encourage the patient to continue with the work. If necessary, phone calls can be made to solve problems, discuss motivation, or simply get in touch with an inactive patient. Patients also have the option of requesting a meeting face-to-face with their therapist if this can be helpful for their further progress in the treatment.

The therapists work at an out-patient secondary care clinic, where they are co-located at least once a week when working with guided ICBT. The rest of their week, they have an ordinary workload in the clinic with face-to-face therapies and other clinical work. The therapists are licensed health personnel from various professions: psychologists, registered nurses, a clinical social worker, and a psychiatrist with the majority having one-year continued education in guided ICBT. They have weekly staff meetings where challenges and successes are discussed, as well as weekly supervision with a specialist psychologist.

### Data collection

We analyzed patient responses to an online survey routinely filled out by participants after they have completed the ICBT program eCoping. The survey consists of 28 items, 22 of which require patients to provide numerical responses on rating scales and 6 of which are open ended. The 28 items cover different aspects of the treatment. Majority of the items assess patients’ evaluations of the treatment as a whole, positive changes and benefits that they experienced, as well as potential adverse effects following treatment. The remaining items evaluate how patients experienced the treatment format, the technical interface, and the contact with their therapist during the treatment. In this study we examined responses to the open-ended question “What did you miss in the contact with your therapist?”

### Analysis

Data was analyzed for themes using a conventional content analysis approach (White and Marsh, 2006). Conventional content analysis is a suitable method when analyzing a large quantity of written data for relevant themes and topics, where the aim is to grasp the participants’ unique experiences as conveyed through their responses (Hsieh and Shannon, 2005). It is also the case that former qualitative investigations of similar survey-based written responses regarding ICBT have used content analysis as analytical tool (Rozental et al., 2015; Hadjistavropoulos et al., 2018). For our analysis, responses were extracted and read thoroughly by all authors to familiarize themselves with the responses and to get an overall sense of the material. When extracting the data, we found a pattern of responses addressing not only what patients missed in regard to the therapist, but also features of both the program and the patients themselves that constituted challenges to engagement. We had not expected this pattern of findings but chose to include these responses in our analysis as they pointed to significant experiences of the ICBT treatment. Furthermore, it became apparent that some participants had conveyed responses relevant to our research question under the heading “additional suggestions and improvements.” We therefore chose to include these in our analysis. In the next step, the first author identified responses relevant to the overarching research question of what participants missed in the contact with the therapist and other obstacles encountered during ICBT. These were scrutinized closely to identify patterns in the material (White and Marsh, 2006). Data were coded by categorizing the individual responses according to the experiences of ICBT they conveyed. Formulations deemed relevant were reduced to units that attempted to capture the meaning of the entire response, such as “missing face-to-face contact” or “wishing that my therapist understood me better.” Discrete meaning units were counted as a single response, making it possible for individual participants to contribute multiple meaning units to the data set. Care was taken to ensure that the coding categories were close enough to what the participants wrote, while at the same time abstracting sufficiently to be able to group together utterances that were seen as thematically related. These condensed meaning codes were subsequently discussed extensively by all three authors and collaboratively developed into larger themes identifying the most salient aspects of participant’s experiences. Throughout the process of analysis, the material was scrutinized repeatedly to keep the themes sufficiently grounded in participants’ responses. For two of the three themes, we organized their content in different discrete sub-themes. These sub-themes each expressed some dimension of what the participants missed from their therapist or from ICBT treatment. The overarching themes were defined with regard to the aspects of the ICBT treatment they addressed - either the patient, the program, or the therapist.

## Results

### Frequency of responding

A total of 579 patients gave in total 608 unique responses to the open-ended questions “What did you miss in the contact with your therapist?” and “Other suggestions and improvements.” An overview of the response categories and frequency of responses is provided in Table 1. Of these, a total of 219 (35.8%) responses were categorized as expressing experiences on the patient’s part that something was missing during treatment and were included in the further analysis.

**Table 1:** Overview of response categories and frequencies of responses.

Response categories	*N* = 608 (%)
Something was seen as missing or lacking	219 (35.8)
Blank answer field	186 (30.5)
Responses declaring that nothing was seen as missing or lacking	114 (18.7)
Positive experiences	82 (13.4)
Response outside focus of analysis	7 (1.1)

### Qualitative content analysis

We identified three main themes, two of which had a set of corresponding sub-themes. The categories were organized along the dimensions of *Therapist-ascribed shortcomings* (with subcategories *Emotional contact, Tailored contact, Pushing, Clarification of therapist role,* and *Continuity*), *Program obstacles* (with subcategories *Meeting face-to-face, Availability and responsiveness, Flexibility with regard to time* and *The right treatment*), and *Self-attributed limitations.* An overview of the categories, subcategories with examples and frequency of responses are provided in Table 2.

**Table 2:** Overview of qualitative categories and subcategories with examples.

Category	Subcategory	Description	Example
Therapist-ascribed shortcomings (*n* = 67)	Emotional contact (*n* = 21)	Experiences of the therapist as lacking in understanding and authenticity	More directly like “how are you?.” I understand that my answers on the tasks probably could have answered this, but I have not really experienced that I had support and companionship, but I guess that was not the purpose either.
	Tailored contact (*n* = 21)	A wish for the therapist to tailor the treatment to the patient’s difficulties or life situations	More personal contact to discuss my particular challenges, and to understand how my anxiety resembles and differs from typical anxiety experiences.
	Pushing (*n* = 12)	A wish for the therapist to be more encouraging or challenging to facilitate engagement	Personally I need a stricter regime to follow, where I have to report my progression in order to do it thoroughly.
	Clarification of therapist role (*n* = 7)	Uncertainty with regard to what the therapist can be expected to assist the patient with	I was unsure of what there was room for in the contact with the therapist.
	Continuity (*n* = 6)	Negative experiences related to treatment breaks and therapist changes	A little unfortunate with a break during the summer - a stop in the communication and the sense that my therapist knew who I was and “got” me.
Program obstacles (*n* = 132)	Meeting face-to-face (*n* = 76)	A wish for increased in-person contact	I missed more contact face-to-face. Not everything is easy to convey through writing or over the phone.
	Availability and responsiveness (*n* = 33)	Preference for greater synchronicity in communication	That the therapist was more available than only 1 day of the week. If I had questions, I had to wait until that 1 day. I finished the first modules quickly, so even if I was ready to move on, I had to wait until that 1 day before I could move on.
	Flexibility with regard to time (*n* = 16)	A wish for more time to complete each module	A little stressful with the time given. Occasionally, there were short time limits to finish up. Maybe it should be considered to let the patient have a little more time on treatment?
	The right treatment (*n* = 7)	Experience of the treatment not being the right fit	The treatment wasn’t right for me.
Self-attributed limitations (*n* = 20)		Perceived lack of intrinsic motivation or engagement	My therapist was very encouraging and answered my questions to the best of her abilities. I think I was the one lacking in terms of shortcomings.

#### Theme one: Therapist-ascribed shortcomings

The theme *Therapist-ascribed shortcomings* contained subcategories where responses related directly to what patients missed from their therapist. The first sub-category *Emotional contact* pertained to experiences that there was something lacking in the emotional bond between patient and therapist. Some wished that their therapist would have shown greater care, understanding and personalized support throughout the treatment. This included suggestions for checking in on the patients’ wellbeing, in addition to making sure time limits were held and the material understood: “For a time I was feeling very much down. My therapist was aware of this. It would’ve been nice if she could have called me then.” Instead, some participants experienced the interaction as focused on task-related feedback in a manner that could feel emotionally detached:

I felt that everything was very impersonal and at times a little unprofessional. I missed some acknowledgement of my feelings and “problems.” Instead, it turned out to be a mechanical follow-up with focus on finishing the program and deadlines.

The theme also encompassed experiences of therapists missing out on the larger picture of patients’ lives and challenges, by focusing too rigidly on the program and its tasks: “I missed a more personalized contact where I could tell a little more about how I am doing, and not just how it is going with the exercises or homework.” Others indicated that they missed authenticity from their therapist, referring to the patient’s experience of their therapist as genuinely invested and emotionally present, as opposed to providing responses that could feel somewhat artificial and thus less convincing:

Feedback from the therapists can be a bit superficial, where I have received the “you are doing good and well” quotes from two different therapists in the beginning of a module, which is supposed to encourage and praise the patient. You can tell that the therapist uses a script to do exactly this. That is also one of the reasons I was not satisfied with the internet treatment. It does not seem sincere.

The subcategory *Tailored contact* summarizes the experiences of patients that wished that their therapist would have flexibly adjusted the therapeutic contact to their personal difficulties and reactions to treatment. Some missed an explicit focus on the idiosyncratic ways symptoms and problems manifested in their life, in contrast to a more generalized understanding: “Solutions that fit *me* and my anxiety, not generally. No one is alike.” This also included preferences for targeting treatment to one’s unique circumstances, such as being out of work or struggling with loneliness. Others wished that the therapist had provided more concrete and specific feedback on the obstacles and challenges they encountered while working on the modules: “Tips for exposing, specific exercises, and ways to narrow areas for exposure. It was difficult to judge whether I progressed too slowly.” The subcategory *Pushing* concerned wishes for therapist behaviors that would have facilitated the patients’ agency to a greater extent. These patients reported that they missed external encouragement to comply with the program, in the form of the therapist motivating them through support, challenges in the exposure exercises, and specific advice to help them follow through with the treatment: “He could’ve pushed me into doing several, and more challenging exposures. It is always easy to take the path of least resistance when things are difficult.” The subcategory *Clarification of therapist role* concerned experiences of lacking elucidation of what patients could expect from therapists. Patients stated that they would have liked a better explanation of the therapist role and the limits this imposed on the therapeutic relationship. These experiences suggest that uncertainty about the matters that are suitable to bring up with the therapist, left the patient withholding questions and concerns that appeared throughout treatment. One patient stated that “I was scared of nagging and imposing on him. I would have liked to ask many more questions, but I was not sure if that was what his job was.” Another would have liked to be provided with examples of what you could contact your therapist about, while yet another reported seeing a psychotherapist in private practice in order to find answers to questions that the patient was unsure about asking the therapist. The final subcategory was *Continuity.* It encompasses the experiences of some patients that longer breaks and unplanned therapist changes during treatment served as obstacles for satisfaction with treatment: “A little difficult to open up fully when you have to change therapists in the middle of the program.” This category sheds light on the frustration experienced when patients lost the emotional bond they had established with their initial therapists, and the sense of having to start over in some way.

#### Theme two: Program obstacles

This overarching theme encompassed responses that concern the format and structural features of the ICBT treatment. The first subcategory, *Meeting face-to-face*, captured wishes for the program to contain more direct and in-person interaction with the therapist. Responses in this category ranged from preferring face-to-face therapy, to suggestions of adding more direct contact to the existing ICBT program and procedure. The majority stated that they would have preferred to meet the therapist once or up to a few times during the treatment in order to talk over status quo, resolve any problems, and increase motivation and engagement:

Sometimes there may be a need to speak with someone in person since it is not always easy to formulate everything [in writing]. Also, it can be important with a consultation along the way to keep pace with the treatment and ensure that it is progressing.

These participants felt that one additional meeting in person, often suggested at the midpoint of treatment, would have been sufficient to facilitate emotional support, understanding of treatment, as well as management of practical issues. Others stated that they would have preferred even more frequent and regular meetings with their therapist throughout the program in addition to the digital interaction on the platform:

I guess I missed weekly conversations either on the phone or at the office; where we could have gone over the modules after I had read them. Asked questions and shared frustrations with things I do not agree with. Then it would have been easier to complete the modules.

A general tendency in these responses suggested that when a patient experienced difficulties with the treatment, they preferred face-to-face contact with their therapist: “During the exposure tasks in difficult situations, I would have preferred a supervisor face-to-face. It was difficult to talk to the message box about the challenges I faced while I was doing the exposures.” Some reasoned that face-to-face contact would have made it more difficult for them to avoid psychological discomfort and anxiety - something they might feel inclined to when progress relied on their own self-directedness with working on the tasks, and not on direct observations by the therapist. Others expressed that they were satisfied by the structure and content of the program but would have preferred face-to-face treatment instead of internet-based treatment. They explicitly expressed that they missed human contact and a sense of synchronicity in the communication with their therapist, seeing this as a prerequisite for interpersonal connection: “I’d rather that it was not online, I missed the more natural conversation you have when you speak in the same room.” A subset of the patients in this subcategory also stated that they would have preferred more direct and in-person contact, without specifying how much contact would have been satisfactory. The subcategory *Availability and responsiveness* denoted the experiences of missing a more available therapist throughout treatment who could check in and answer questions while working through the modules. These responses expressed dissatisfaction with the asynchronicity or time-lag in the communication schedule of the program, and a wish for increased flexibility with regard to patient queries and therapist responses. Several patients stated that it was impractical having to wait several days to get an answer to a question or to be checked off to the next module. Patients that appeared to struggle with making contact with their therapist stated that increased availability would have lowered the threshold to reach out for help and guidance.

I think I would have contacted my therapist if I could get feedback during the evening. I had to be completely stuck and not able to move forward at all before I would make contact. That said, the answers I received the times I did make contact were good and helped me back on the right track and put things in perspective.

The subcategory *Flexibility with regard to time* concerned requests for a less rigid temporal schedule to the program. These patients reported that they struggled to complete the modules and related tasks within the expected time frame and missed flexibility from their therapist with regard to making deadlines. Several of these responses emphasized the difficulty of combining a challenging treatment with the demands of everyday life.

I know that treatment is supposed to take place over a certain period of time, but in particular situations pressure from work, family situation, health, can affect how much you can work with this and then it should be possible to have more time.

Others described how increased flexibility with regard to time would have provided an opportunity to thoroughly learn the material and integrate it into daily life before moving on to the next module: “To me it was too short to only use one week with each module. To work well with strategies and practice so they “stick,” I would have preferred two weeks.” The final subcategory is called *The right treatment*, with responses denoting experiences of the program or treatment modality as ill-fitting or unhelpful in general.

#### Theme three: Self-attributed limitations

Responses in this category emphasized that something was lacking on the patients’ part in terms of intrinsic motivation, self-discipline, or engagement to work with the modules or ask the therapist for support. Responses ranged from experiencing depressive inaction to milder degrees of self-attributed motivational deficits. In these instances, having to explicitly ask for support appears to be a barrier to reach out for help: “I should have been better at making contact. I encountered problems and challenges on the way. I find it difficult to turn to others in those situations.” Some stated that they had a difficult time completing treatment because they lacked intrinsic motivation or commitment to follow through with the program:

Quitting now lies with me, and no one else. I need to motivate myself more. It was too tough for me, but I am not planning to have panic anxiety for the rest of my life so I would like to try again another time.

Some of the responses expressed a tendency to attribute lack of progress in the treatment to themselves and their own effort. In several responses, there was an element of self-blame like the following patient notes:

Looking back, I should have reached out when I thought it was difficult to follow through. I should have been clear about being stressed and not being able to find the time, that it was too much, so it was mostly my own fault.

Others expressed that they could have used the opportunity to ask their therapist more questions or that they could have been more active throughout treatment.

## Discussion

The main aim of the current study was to investigate patient experiences of the contact with their therapist during guided ICBT in routine care. We used qualitative content analysis to explore patients’ experiences of what they missed in the contact with their therapist after completing guided ICBT for MDD, SAD and PD. Of 579 patients that received the survey, 608 unique responses were provided on the open-ended questions. Of these, 219 responses gave voice to some degree of perceived lack or limitation in their interaction with the therapist or the treatment in general and were subsequently analyzed. Three main themes emerged in the content analysis: Therapist-ascribed shortcomings (*n* = 67), Program obstacles (*n* = 132) and Self-attributed limitations (*n* = 20).

The first theme in our material, *Therapist-ascribed shortcomings*, concerned experiences of something missing or lacking in the contact with the ICBT therapist during treatment. Some expressed wishes that their therapist had been more understanding and authentic, or more responsive to them as persons. There was also a perception by some that the responses of therapists were scripted or artificial. Patients thus gave voice to perceptions of insufficient relational or emotional depth in the relationship. Others wished that the therapy had been better tailored to their particular difficulties, and some wished the therapist had facilitated motivation by pushing and challenging them more. A few patients described that they were not sure what the therapist’s role was, and a few others expressed dissatisfaction with the breaks and therapist changes that took place during treatment. Overall, the responses in this category shed light on the importance of the presence of a supportive and engaged therapist in guided ICBT. This is in line with the meta-synthesis by Patel et al. (2020) which identified that in the majority of studies analyzed, patients expressed the value of personal and human contact in treatment, and this appeared to lack in therapies with less favorable outcomes. Furthermore, Holländare et al. (2016) found an association between the amount that therapists affirmed their patients in guided ICBT, and the patients adherence to and symptom improvement from treatment. In a qualitative study on patient experiences of guided ICBT, Johansson et al. (2015) found that patients who experienced their therapist as not caring, were less engaged during treatment. The opposite direction of this relationship has also been described. For example, Bendelin et al. (2011) describe how patients who were less engaged experienced themselves to be more impaired by the lack of support from their therapist. It can be hypothesized that patients that struggle with the structural features and format of the treatment, usually require more support from their therapist and vice versa; patients who appear to intuitively engage with the program and tasks at hand, may need less support.

Overall, the patients who expressed that something was missing in the contact with their therapist constituted a small part of the responses in the sample, even after being directly asked. This could be seen as supporting the findings that a collaborative and supportive relationship is possible within this treatment modality, despite communication and contact being of a more limited nature (Pihlaja et al., 2018). As noted by Probst et al. (2019), the reduction of interactive channels in ICBT does not necessarily impoverish communication, as participants either find ways to compensate for these limitations or may even see them as an advantage of the treatment format. It is likely that participants who experience ICBT in this manner are prone to associate the format with sufficient support, and thus as enabling autonomy, self-efficacy and self-directed therapeutic activities in the manner pointed out in other qualitative studies (Bendelin et al., 2011; Patel et al., 2020). The themes that emerged are nevertheless important as they point to significant experiences of being inadequately understood, validated, guided, or supported that could affect treatment engagement. Consequently, it is of relevance to explore further how ICBT therapists may convey sufficient levels of empathy, care and authentic responding within the confines of the limited communication format of this treatment modality.

The second theme, *Program obstacles*, concerned experiences of barriers to engagement through the way the ICBT program organizes interaction between patient and therapist with regards to mode, synchronicity, frequency of contact and the imposed deadlines. Patients in this category expressed a wish for increased face-to-face contact, that their therapist was more available throughout the week and would respond more swiftly, and that they had the option to ask for extended deadlines for completing program modules. The individual preferences for how this should be carried out varied greatly. Some patients saw the program as a reasonable fit but would have liked it augmented by one or two in-person meetings that could make it easier to clarify issues or make sure they were on the right track. Others expressed that they would have liked even more frequent meetings, perhaps more in line with a “blended” treatment format. A minority stated that they would have preferred face-to-face treatment instead of ICBT. The results from this category illuminate the individual preferences of patients receiving guided ICBT, which have been described in previous studies (Patel et al., 2020). The idea that patients prefer different types of guidance in ICBT suggests that identifying patients who benefit from treatment without much assistance, can free up time and resources to aid the group of patients that may need frequent interaction and adjustment of the program structure.

Across qualitative studies conducted on patient experiences of ICBT, it is a recurrent theme that some patients are less satisfied with the structural features of the online treatment format. Patients in a study by Hadjistavropoulos et al. (2018) describe experiences of the treatment program as inflexible, while patients in studies by Johansson et al. (2015) and Holst et al. (2017) reported that they wished treatment consisted of increased face-to-face-treatment. The present study adds to this pattern of findings. Although it may seem useful to individually adjust the treatment program to meet patients’ wishes and preferences, research on factors that affect outcome in ICBT indicate that not all means of introducing flexibility necessarily make treatment more effective. For instance, investigations of guidance modality have not yielded differences between contact that is synchronous, meaning that the messages are exchanged in real time over the phone, and contact that is asynchronous, meaning contact that is provided with some delay in time as through emails or on a forum (Titov et al., 2009; Lindner et al., 2014). Several studies have explored whether receiving therapist guidance more than once a week makes a difference for patients participating in ICBT for different mental disorders. Generally, there is no indication that increased contact frequency or response time matters for outcome, and it seems that ICBT with less therapist contact can be effective (Klein et al., 2009; Aardoom et al., 2016; Hadjistavropoulos et al., 2020). The finding that ICBT can be equally effective with different types of support and guidance, indicates that what patients assume would be helpful might not necessarily make a notable difference. There thus seems to be a divergent pattern of findings between quantitative investigations with a larger number of participants, and qualitative investigations where the individual experiences are explored. It is therefore not clear how the research literature should inform clinical practice when it comes to the role of flexibility, contact frequency and responsivity in guided ICBT. It may be that it is the content and quality of therapist contact that affects how the guidance in ICBT is experienced, more than the quantity of contact. For example, in the current study several patients explicitly stated that they missed meeting their therapist in person. However, it does not necessarily seem that it is the meeting itself that matters - some of the replies give the impression that face-to-face meetings allow for a closer emotional relationship and greater support from the therapist. In this sense, requests for increased frequency or flexibility with regard to contact can potentially be seen as proxies for a relational depth that some participants miss. Although we cannot know for certain based on our material what motivates patients to ask for increased face-to-face contact with their therapist, we can hypothesize that some of them actually miss a stronger emotional bond. Although the relevant dimensions of support have proven difficult to identify through large-scale quantitative analyses, it remains of interest to understand better how ICBT gives rise to divergent participant experiences, and how program features may be adjusted to facilitate adherence for those who currently see the format as less satisfying.

The third theme, *Self-attributed limitations*, concerned patients’ experience of being responsible for their difficulties in motivation and engagement. Several of the patients in this category attributed experienced difficulties with the program to their own effort and commitment to the therapy. Some went further in stating that it was their own fault that the therapy failed. The idea that a lack of motivation can affect psychotherapy outcomes is well documented in the psychotherapy literature (Zuroff et al., 2007; Krebs et al., 2019). In a therapeutic context, motivational work can refer to the patient taking an active part in their own recovery and wanting change in their life for their own sake. This category also included a group of statements from patients who appeared to blame themselves, implying that there was something with them as a person rather than the treatment or the contact with the therapist that could explain the difficulties of completing treatment. These patients also indicate through their responses that they hesitated to reach out to their therapists to address their struggles. As such, they resemble the group of “Strivers” conceptualized by Bendelin et al. (2011), who are characterized by their ambivalence towards the working process of ICBT and experiences of missing support from their therapist throughout treatment. This is in line with prior qualitative studies that report a pattern of some patients experiencing decreased engagement (Patel et al., 2020). Although patients in the eCoping program have the option of asking their therapist for additional support or a meeting face-to-face if they experience difficulties, some patients indicated that they had a high threshold for using this option. This is in line with studies conducted on scheduled versus optional guidance that indicate that few patients ask for additional guidance if it is up to themselves to reach out for help (Hadjistavropoulos et al., 2017; Zetterberg et al., 2019). The self-attributed limitations expressed by the patients in our study may represent a small but important group of patients that need additional support and monitoring throughout their therapeutic process. A central task of guided ICBT therapists then, might be to develop the ability to discern patients that struggle with the format and tasks from those who do not, to provide adequate guidance and support. Prior research on therapist behaviors in ICBT has found that supportive interventions are positively related to outcome (Paxling et al., 2013; Holländare et al., 2016). The therapist can play a central role in fostering motivation in the patient through validation and empathy which at the same can counter unproductive blaming and self-deprecation.

It may be the case that the novel format of ICBT challenges the way we understand and conceptualize the therapeutic alliance in this treatment modality. One reconceptualization is the tripartite therapeutic alliance between patient, therapist and treatment program as suggested by Cavanagh et al. (2018). The three overarching categories of responses that were identified in the current study seem to correspond to the patient, therapist, and program dimensions of Cavanagh and her colleagues’ conceptualization. With a reformulation of the therapeutic alliance in guided ICBT follows the suggestion that the format may pose new challenges to therapists that train for and work with guided ICBT.

## Reflexivity

A common challenge with the conventional content approach may be that themes are imprecise or important themes fail to be detected, thus compromising the credibility, or internal validity, within the material (Hsieh and Shannon, 2005). To counter this, the current team of researchers throughout our analytic procedure employed a team-based reflexive stance, striving to be aware of how our own perspectives and presuppositions may influence our interpretation of the material. This was useful, as we had different connections to the guided ICBT treatment program. The third author has led the implementation and evaluation of the program, while the first author had little prior experience with it. The second author had previously been involved in qualitative research on other aspects of the guided ICBT program, but did not have close experience with the content, organization, and practical clinical implementation of it. Our acknowledgment and continual discussion of our different points of view helped us maintain a reflective distance to the data, as well as using our perspectives to highlight different aspects of the material. Furthermore, the results of the current analysis were presented for a researcher external to the project with extensive experience with qualitative methodology. This researcher surveyed the thematic structure as a critical auditor, assessing the validity of the categories and themes with regard to how well they reflected the coded data. Another challenge is that the limited nature of the written material prevents theory building or making inferences about the nuances of lived experience, in contrast to data based on in-depth interviews (Hsieh and Shannon, 2005). LaDonna et al. (2018) argue that responses to open-ended questions are unlikely to support rigorous qualitative insight due to their brief nature. Specifically, these authors point to lack of data richness as responses often do not convey contextual information, nuances, layers of detail or complex patterns of relational and personal meaning (LaDonna et al., 2018). This was the case for the current material, as many of the responses were relatively short and sometimes ambiguous in their formulation. The researchers tried to always be aware of this, and care was taken to preserve the patient’s experiences as best as possible. We also aimed for analytical rigor through a process of iterative and reflexive analysis of the data, and by having the thematic structure critically audited.

## Clinical implications

Based on the results from the current study, some clinical implications can be delineated that may aid ICBT therapists in their clinical training and supervision. Training therapists to identify patients that struggle with motivation and who can benefit from monitoring, guidance, and support, can free up time and space from those who do not. Motivational work in guided ICBT can include clarifying the patient’s expectations of treatment and exploring their perceptions of possible benefits and disadvantages of participating in treatment. Further, it can be helpful to identify areas in advance that might pose a particular challenge for the patient, such as reaching out for help when experiencing difficulties, doing homework within the deadlines, or plan and conduct exposure exercises. The therapists will be important in stimulating patients’ reflection on these topics, and in finding possible solutions to help the patient stay on course during treatment. Tailoring the therapeutic contact can increase patients’ experienced relevance and benefit from treatment without having to adjust the structural features of the program.

Another possible implication regards the training of future ICBT therapists. The results from the current study illuminates the importance of therapists´ ability to provide individualized and tailored support to a range of patients with different expectations and inclinations to engage in treatment. This may require a particular emphasis on the fostering of therapeutic sensitivity and flexibility in novel ICBT therapists. It is of relevance whether therapists have the capability to practice skills that makes it possible to be experienced as sufficiently attuned and genuinely interested in the patient as a person within the existing ICBT framework for communication and interaction. This might entail being more attentive to the particular life circumstances of the patient, and how this might impact on his or her challenges. Feeling seen and understood in this manner could provide important validation that may assist patients in engaging more actively with the program. It may also be useful to train therapists to be specific in their feedback to patients, for instance by being explicit on why the therapist sees the patient as having performed tasks adequately. If not, it is a risk that more generic responses will be seen as less genuine and less appreciative of the hard work patients are engaging in.

## Limitations

The current study had several limitations that ought to be taken into consideration. Firstly, the patients recruited had three different psychiatric disorders. It is possible that different psychiatric conditions require different therapeutic approaches in guided ICBT, and that some therapist behaviors or skills will be more or less important when treating different conditions. However, this could also be seen as a strength in that we may be able to delineate some general patterns of experiences that are central in guided ICBT across three highly prevalent psychiatric conditions. Second, this study did not allow for exploring the association between the responses and the patient outcomes, which could have been useful in determining whether negative experiences of treatment actually were associated with poorer outcomes. A third limitation was that responses were provided after treatment was completed, which makes the results susceptible to bias in that patients may only provide the experiences that they remember the best or thought of more recently. Lastly, it ought to be considered that patients may feel ambivalent about admitting negative aspects of the relationship with their therapist, as it can be conflicting to provide negative evaluations about someone who helped you and you feel thankful for.

## Conclusion

The current study explored what patients missed in the contact with their therapist during guided ICBT for major depressive disorder, social anxiety disorder and panic disorder in routine care. Although patients were explicitly asked about what they missed in the contact, only 219 expressed such responses, including an array of experiences that also regarded other aspects of treatment. Three main themes emerged in the content analysis. The first theme, Therapist-ascribed shortcomings, concerned experiences of missing closer contact with the ICBT therapist, either through a nearer emotional bond or through an increased focus on the patient’s particular challenges and needs in treatment. The second theme, Program obstacles, regarded perceived hindrances in how the eCoping program structures interaction and responsivity. The third and final theme, Self-attributed limitations, concerned patients’ experience of being responsible for their difficulties in motivation and engagement.

The findings of the study illuminate the diverging needs, motivations and expectations of patients that receive guided ICBT in routine care. Where some patients experienced innate motivation and readily engaged with the treatment program, others struggled to maintain progress, and appeared to be at risk of dropping out of treatment. Patients also varied with regard to how they perceived flexibility within the internet-delivered treatment program, where many missed the option of receiving more guidance and support from the therapist either more frequently throughout treatment, or through increased physical contact in addition to the contact provided through the eCoping program. The role of the therapist in providing human contact and support appeared as central, especially with patients that experienced the treatment format as particularly challenging or inflexible. The results from the study add to the qualitative literature on patient experiences in guided ICBT and illuminate the importance of exploring the emotional and relational experiences of participants in internet-delivered treatments. Future research should explore whether the themes that appeared in the current analysis are relevant across patient populations, age groups, clinical settings and digital formats such as virtual reality or chat bots. Additionally, future research should attempt to identify early signs and characteristics of patients that struggle with maintaining progress and are at risk of dropping out of guided ICBT. This knowledge can help therapists provide high-quality tailored support that may help them back on the right track.

## Data availability statement

The original contributions presented in the study are included in the article/supplementary material, further inquiries can be directed to the corresponding authors.

## Ethics statement

The Western Regional Committee for Medical and Health Research Ethics in Norway approved the present study (2012/2211/REK;2015/878/REK). All included participants signed a written informed consent form.

## Author contributions

TN is the primary investigator in the project and responsible for the data collection. The current analysis was conceived, designed and performed by all three authors. The three authors also equally contributed to the writing of the paper. All authors contributed to the article and approved the submitted version.

## Conflict of interest

The authors declare that the research was conducted in the absence of any commercial or financial relationships that could be construed as a potential conflict of interest.

## Publisher’s note

All claims expressed in this article are solely those of the authors and do not necessarily represent those of their affiliated organizations, or those of the publisher, the editors and the reviewers. Any product that may be evaluated in this article, or claim that may be made by its manufacturer, is not guaranteed or endorsed by the publisher.
